# HIPK2 and extrachromosomal histone H2B are separately recruited by Aurora-B for cytokinesis

**DOI:** 10.1038/s41388-018-0191-6

**Published:** 2018-03-22

**Authors:** Laura Monteonofrio, Davide Valente, Manuela Ferrara, Serena Camerini, Roberta Miscione, Marco Crescenzi, Cinzia Rinaldo, Silvia Soddu

**Affiliations:** 1 Unit of Cellular Networks and Molecular Therapeutic Targets, Regina Elena National Cancer Institute—IRCCS, Rome, 00144 Italy; 2 Institute of Molecular Biology and Pathology (IBPM), National Research Council (CNR), c/o Sapienza University, Rome, 00185 Italy; 3 Core Facilities, Italian National Institute of Health, Rome, 00161 Italy

## Abstract

Cytokinesis, the final phase of cell division, is necessary to form two distinct daughter cells with correct distribution of genomic and cytoplasmic materials. Its failure provokes genetically unstable states, such as tetraploidization and polyploidization, which can contribute to tumorigenesis. Aurora-B kinase controls multiple cytokinetic events, from chromosome condensation to abscission when the midbody is severed. We have previously shown that HIPK2, a kinase involved in DNA damage response and development, localizes at the midbody and contributes to abscission by phosphorylating extrachromosomal histone H2B at Ser14. Of relevance, HIPK2-defective cells do not phosphorylate H2B and do not successfully complete cytokinesis leading to accumulation of binucleated cells, chromosomal instability, and increased tumorigenicity. However, how HIPK2 and H2B are recruited to the midbody during cytokinesis is still unknown. Here, we show that regardless of their direct (H2B) and indirect (HIPK2) binding of chromosomal DNA, both H2B and HIPK2 localize at the midbody independently of nucleic acids. Instead, by using mitotic kinase-specific inhibitors in a spatio-temporal regulated manner, we found that Aurora-B kinase activity is required to recruit both HIPK2 and H2B to the midbody. Molecular characterization showed that Aurora-B directly binds and phosphorylates H2B at Ser32 while indirectly recruits HIPK2 through the central spindle components MgcRacGAP and PRC1. Thus, among different cytokinetic functions, Aurora-B separately recruits HIPK2 and H2B to the midbody and these activities contribute to faithful cytokinesis.

## Introduction

Up to one-third of human cancers are likely to originate through unscheduled tetraploidization, a genetically unstable state that can promote aneuploidy and chromosomal instability (CIN). Faithful cytokinesis is required to preserve ploidy and prevent such genetically unstable state [1–3]. Cytokinesis proceeds through different phases starting from specification of the cleavage plane and ingression of cleavage furrow, progressing to central spindle assembly and subsequent midbody formation, ultimately ending with abscission [4–6]. The right execution of each phase strictly depends on the success of the previous one, thus chemical biology approaches have been developed to spatially and temporally probe the different phases [7].

Aurora-B is a Ser/Thr kinase that in mammals was originally identified as a kinase overexpressed in cancers [8] and required for cytokinesis [9]. Along with key roles in histone H3 phosphorylation, chromosome condensation/alignment, and spindle assembly checkpoint in mitosis, Aurora-B acts at different steps throughout cytokinesis [10, 11]. In a spatio-temporal manner, Aurora-B promotes the formation of cleavage furrow, central spindle, and midbody by phosphorylation and recruitment of motors and microtubule-associated proteins, including the centralspindlin components MKLP1 and MgcRacGAP, the Rho GTPase activator ECT2, and the microtubule-bundling protein PRC1 [12–15]. Finally, when lagging chromatin is present at midbody, Aurora-B prevents abscission through activation of the abscission checkpoint [16, 17].

The midbody is a tightly packed antiparallel microtubule bridge that transiently connects the daughter cells at the end of cytokinesis. It serves as a platform to orchestrate cytoskeleton rearrangements, plasma membrane remodeling, and recruitment of the functional complexes needed for abscission. During its formation, several proteins relocate from central spindle to distinct midbody domains [18]. Besides Aurora-B, midbody assembly and function is regulated by the mitotic kinases CDK1, PLK1, and Citron kinase, which are crucial for localization, interaction, and enzymatic activity of several cytokinesis factors [4]. Recently, we have described the contribution of an additional kinase, homeodomain-interacting protein kinase 2 (HIPK2), and its phosphorylation target, the extrachromosomal histone H2B, in the control of midbody abscission and in prevention of tetraploidization and CIN [19, 20].

HIPK2 is a Tyr-regulated Ser/Thr kinase [21, 22] involved in DNA damage response (DDR) and development [23–25]. In interphase, HIPK2 mostly localizes at nuclear speckles [26] and its nuclear activity is relevant for anticancer therapy because it induces p53-dependent and -independent apoptosis in response to cytotoxic drugs [27, 28]. Histones are the nucleosome assembly proteins; however, a few extrachromosomal activities of histones have been described [29, 30]. In cytokinesis, HIPK2 and extrachromosomal histone H2B colocalize at midbody independently of the presence of DNA, such as chromosome bridges, lagging chromatin, or ultra-fine BLM bridges [19]. At midbody, HIPK2 phosphorylates H2B at Ser14 (H2B-S14^P^) and contributes to abscission [19]. We also showed that H2B localizes at midbody independently of HIPK2, but the absence of the kinase results in loss of H2B-S14^P^, impaired abscission, and accumulation of tetraploid and polyploid cells that contribute to CIN and increased tumorigenicity [19, 20]. Of relevance, the sole expression of a phosphomimetic H2B-S14D mutant in HIPK2-null cells abolishes cytokinesis defects, restores cell division and proliferation [19], and inhibits tumorigenicity [20]. These data show that HIPK2 controls cytokinesis through extrachromosomal H2B-S14^P^ and this activity is linked to tumorigenicity. However, which are the molecular pathways involved in the recruitment of HIPK2 and H2B to midbody is still unknown.

In this study, we evaluated the possible contribution of RNA and mitotic kinases in the midbody recruitment of HIPK2 and H2B. We found that both proteins are separately recruited through the activity of the same kinase, Aurora-B.

## Results

### H2B and HIPK2 localize at the midbody independently of RNA

**Fig. 1:**
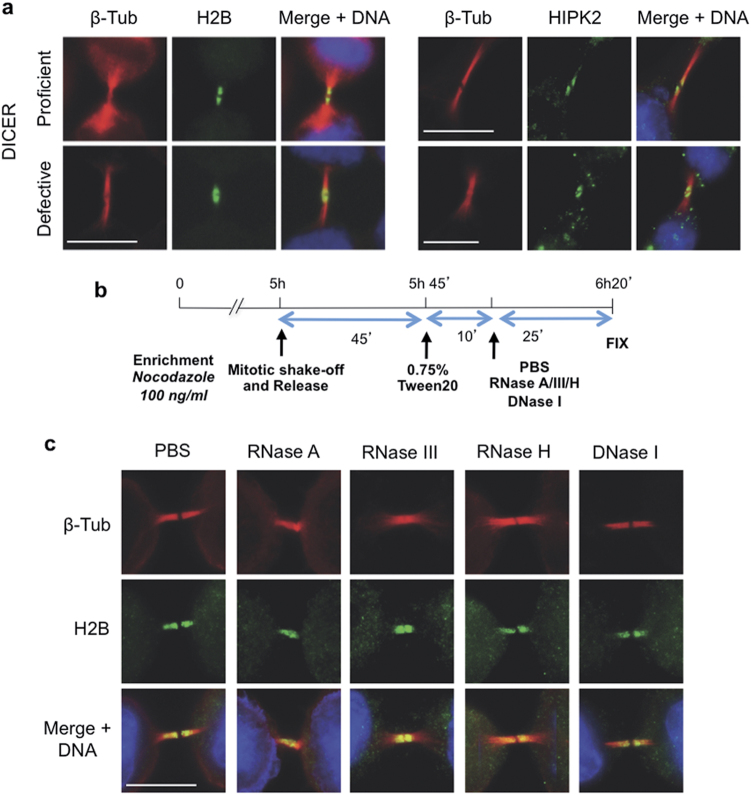
H2B and HIPK2 localize at midbody independently of RNA. **a** Representative images of proliferating HCT116 DICER-proficient and -defective cells analyzed for midbody localization of histone H2B (green; left panels) and HIPK2 (green, right panels). Midbodies were marked with anti-β-tubulin Ab (β-Tub, red); DNA was visualized with Hoechst (blue). **b** Schematic representation of HeLa cells telophase enrichment with nocodazole, permeabilization, and treatment with RNase A, RNase III, and RNase H to cleave, respectively, single-strand RNA, double-strand RNA, and RNA/DNA hybrid. PBS and DNase I were used as negative controls. **c** After treatment, cells were fixed and stained with anti-β-tubulin Ab (red) and anti-H2B (green). DNA was marked with Hoechst (blue). Representative images of HeLa cells treated with the indicated enzymes. Each midbody visualized (*n* > 50 in two independent experiments) was positive for H2B staining. Scale bar is 10 μm

Previous studies have shown that RNA is required for the assembly of protein complexes such as the 53BP1 foci in DDR [31]. Thus, we tested the possibility that H2B and/or HIPK2 midbody localization might depend on the presence of RNA. We first evaluated whether microRNAs might contribute to midbody localization. Double immunofluorescence (IF) for the midbody marker β-tubulin and H2B or HIPK2 [19] were performed on parental HCT116 cells and their DICER-defective derivatives showing decrease amount of microRNAs [32]. No apparent difference was detectable between the two cell types regarding the midbody localization of both H2B and HIPK2 (Fig. 1a), indicating that microRNAs are dispensable for this event.

To assess whether other types of RNAs might be involved, we treated live HeLa cells with RNases adapting the protocol from Francia and colleagues [31] (Supplementary Figure S1a). We treated live cells enriched in ana-telophase to assess midbody formation and protein localization in the presence of different types of RNases targeting single and double-strand RNA, and RNA/DNA hybrid (Fig. 1b). As H2B and HIPK2 localize at the midbody independently of DNA [19], DNase I treatment was used as negative control together with buffer-treated cells. As assessed by IF, none of these treatments was able to inhibit midbody formation or the midbody localization of H2B (Fig. 1c) and HIPK2 (Supplementary Figure S1b), indicating that RNAs are dispensable for their midbody recruitment.

### Aurora-B kinase activity is required for midbody localization of HIPK2 and H2B

**Fig. 2:**
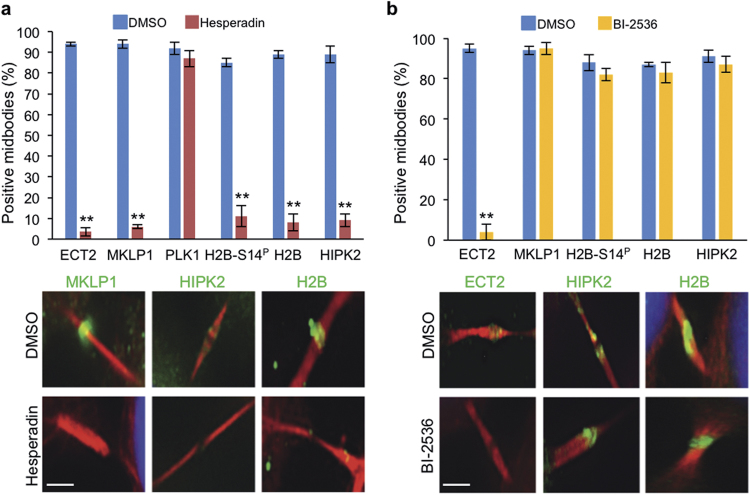
The kinase activity of Aurora-B, but not of PLK1, is required for midbody localization of HIPK2 and H2B. **a**, **b** Unsynchronized HeLa cells were treated with 100 nM Hesperadin for 80 min or with 1 µM Bi 2536 for 20 min; solvent DMSO was used as control. After treatment, cells were fixed and stained with the indicated Abs (green) in combination with anti-β-tubulin Ab (red). DNA was visualized with Hoechst (blue). The percentages of midbodies positive for the indicated proteins were reported as mean ± SD of three independent experiments. Representative images of indicated staining at the midbody are reported below each chart. **p < 0.001. Scale bar is 1 μm

To further investigate the mechanisms involved in HIPK2 and H2B midbody recruitment, we started with inhibiting two of the master kinases involved in cytokinesis and midbody formation, PLK1 and Aurora-B. To avoid early-stage effects on central spindle formation, PLK1 and Aurora-B were inhibited by BI 2536 and Hesperadin, respectively, in temporally controlled modes [18, 33] (Supplementary Figures S2a-b). In such conditions, the inhibition of Aurora-B impairs the localization of both HIPK2 and H2B (Fig. 2a), whereas the block of PLK1 does not affect the midbody recruitment of either of the two proteins (Fig. 2b) or their colocalization with Aurora-B (Supplementary Figure S2c). Similar results were obtained with another Aurora-B inhibitor, the ZM-447439 (Supplementary Figure S2d-f) excluding possible unspecific effects of chemical compounds. Thus, although H2B may localize at the midbody independently of HIPK2 [19], these data indicate that midbody recruitment of either proteins requires the same kinase, Aurora-B.

### Aurora-B binds and phosphorylates H2B but not HIPK2

**Fig. 3:**
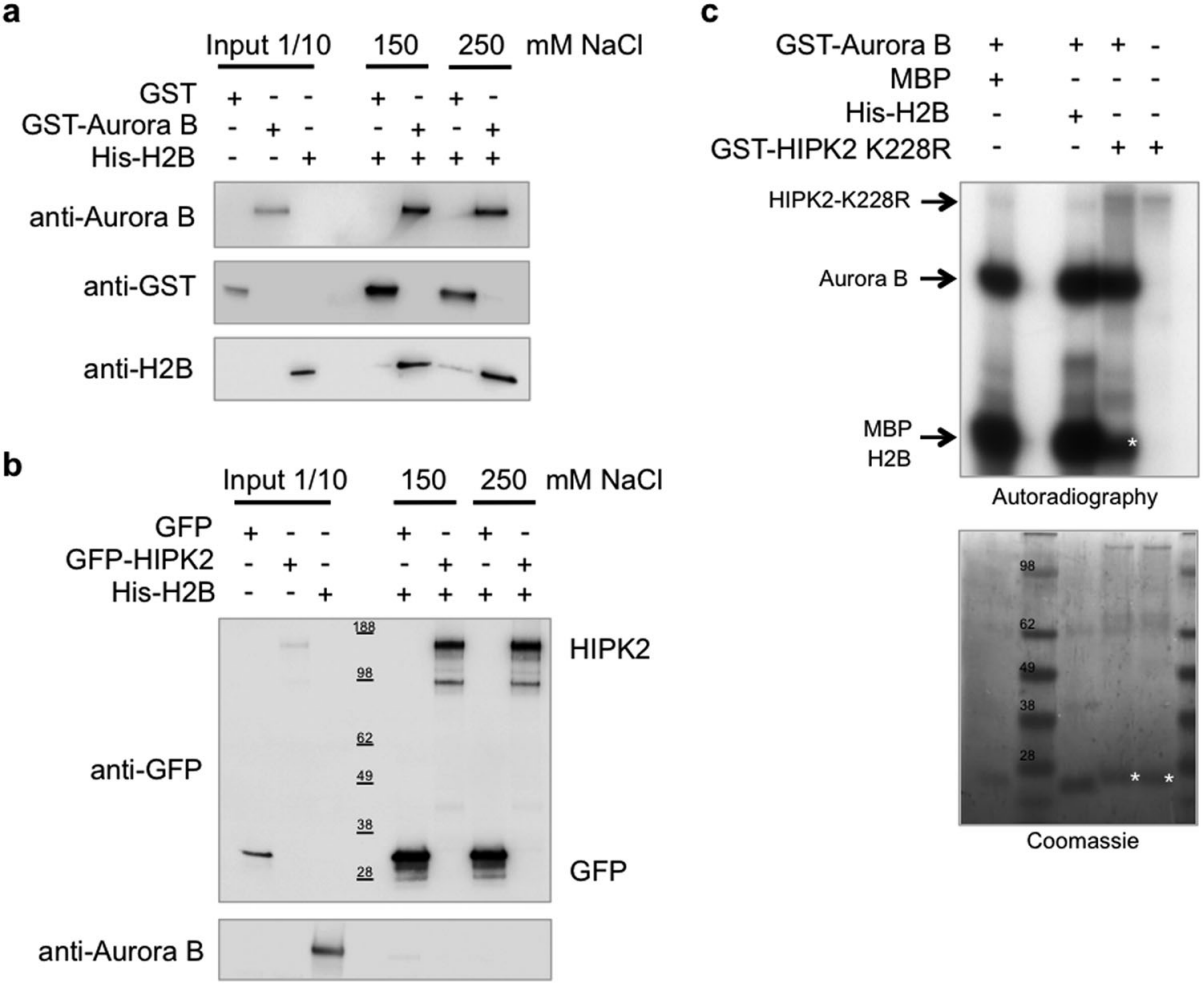
Aurora-B directly binds and phosphorilates H2B. **a** Recombinant His-H2B was incubated in the presence of recombinant GST-Aurora-B or GST alone at two different salt concentrations. Proteins were detected by Western blotting (WB) with the indicated Abs after GST-pull-down of two independent experiment. **b** GFP or GFP-HIPK2 were incubated with recombinant GST-Aurora-B at the same conditions described above. Proteins were detected by WB with the indicated Abs after GFP-immunoprecipitation of two independent experiment. **c** In vitro kinase assays were performed with GST-Aurora-B, as enzymatic source, and His-H2B or GST-HIPK2-K228R as substrates. Myelin basic protein (MBP) was used as positive control. Proteins were separated by SDS-PAGE and radioactivity detected by autoradiography. Coomassie stain was performed as loading control. *n* = three independent experiment. White asterisks mark nonspecific bands

We previously defined the midbody localization of HIPK2 and H2B-S14^P^ also based on their colocalization with Aurora-B [19]. To evaluate whether these colocalizations and the recruitment activity of Aurora-B depend on its direct interaction with HIPK2 and H2B, we performed in vitro binding assays with purified proteins. We found that recombinant glutathione S-transferase (GST)-Aurora-B binds His-H2B (Fig. 3a) but does not directly interact with GFP-HIPK2 (Fig. 3b).

Based on in silico analyses, both HIPK2 and H2B contain putative Aurora-B phosphorylation consensus sequences, thus we performed in vitro kinase assays by using Aurora-B as enzymatic source and purified HIPK2 or H2B as substrates. As HIPK2 possess auto-phosphorylating activity, the kinase-defective HIPK2-K228R mutant was employed. As shown in Fig. 3c, Aurora-B phosphorylates H2B but not HIPK2, suggesting that Aurora-B recruits HIPK2 and H2B through different mechanisms.

Although we cannot completely exclude the possibility that Aurora-B might directly phosphorylate HIPK2 only after its autophosphorylation [21, 22], we know that the kinase-defective HIPK2-K228R mutant can localize at midbody (see below). We also observed that HIPK2 still localizes at midbody upon depletion of extrachromosomal H2B (LM unpublished data) indicating that midbody recruitment of HIPK2 is independent of H2B, further supporting the separate recruitment of the two proteins.

### Aurora-B phosphorylates H2B at Ser32

**Fig. 4:**
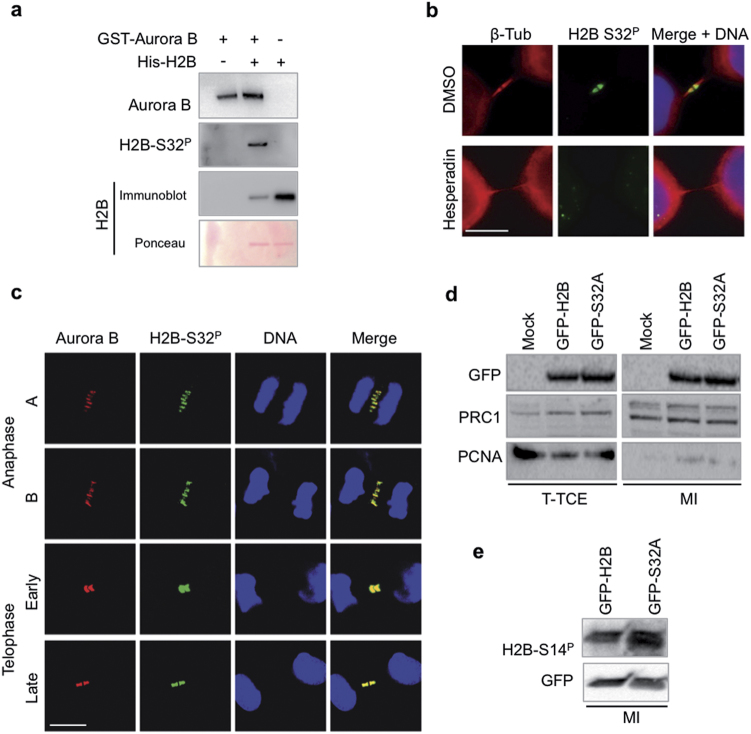
Aurora-B phosphorylates H2B at Ser32. **a** Cold in vitro kinase assays were performed and H2B-S32^P^ detected using anti-p-Histone H2B-Ser32 Ab. Ponceau staining and immunoblot with anti-H2B Ab were used as loading controls. *n* = three independent experiment. **b** Unsynchronized HeLa cells were treated with Hesperadin or DMSO for 80 min and stained with anti-p-Histone H2B-S32 and anti-β-tubulin Abs. Hoechst was used to stain DNA (blue). Representative images of cells at telophase stage are reported. Scale bar is 5 μm. **c** Representative confocal images of HeLa cells at indicated stages of mitosis and cytokinesis showing colocalization between H2B-S32^P^ (green) and Aurora-B (red) *n* = four independent experiment. DNA was stained with Red-Dot2 far-red (pseudo-colored blue); scale bar is 10 μm. **d**,** e** HeLa cells stably expressing GFP-H2B WT or its derivative GFP-H2B-S32A at passage two after the establishment of stable populations (i.e., 15 days after blastacidin treatment) were enriched in telophase and protein lysates were obtained from TCEs (T-TCE) or after midbody isolation (MI). Untrasfected HeLa cells were used as negative control. The indicated proteins were analyzed by WB. Immunoblots with anti-PRC1 and anti-p-Histone H2B-S14 Abs were used as positive control to verify the quality of midbody isolation. Immunoblot with anti-PCNA Ab was used as control to evaluate nuclear contamination. Representative WBs are shown. In **d**, samples were loaded on the same gel and processed on the same filter. Blot was vertically cropped to eliminate non-related samples

H2B contains nine putative Aurora-B phosphorylation sites. In order to identify specific phosphorylation site/s, we analyzed by mass spectrometry (MS) a recombinant His-H2B protein that had been phosphorylated in vitro by Aurora-B. On a total of 126 amino acids, we were able to properly map the H2B region downstream amino acid 35, where, however, we could not detect any phosphorylated Ser or Thr residues (Supplementary Figure S3a). Unfortunately, the N-terminal region encompassing amino acids 1–35 could not be resolved, despite the employment of several cleavage patterns, probably due to the presence of a high positive charge. Nonetheless, H2B-Ser32 phosphorylation (H2B-Ser32^P^) has been already reported and a validated anti-p-Histone H2B-S32 Ab is commercially available [34]. Immunoblot analyses of cold kinase assays performed with recombinant His-H2B and GST-Aurora-B using this Ab showed that Aurora-B is indeed able to phosphorylate H2B-Ser32 in vitro (Fig. 4a).

Next, we verified the specificity of this anti-p-Histone H2B-S32 Ab in IF by using HeLa cells treated with Hesperadin to impair H2B midbody recruitment (Fig. 2a). A clear immunostaining was detected in the midbodies of control cells while no fluorescence was present in Hesperadin-treated cells (Fig. 4b) attesting the specificity of this immunostaining. Therefore, we analyzed the localization of H2B-Ser32^P^ and its relationship with Aurora-B. We found a clear H2B-Ser32^P^ signal already in anaphase A that persists and colocalizes with Aurora-B throughout cytokinesis (Fig. 4c, Supplementary Figure S3b). Of relevance, this phosphorylation was not detectable on nucleosomal H2B belonging to chromosome bridges (Supplementary Figure S3c), supporting a role of extrachromosomal H2B-Ser32^P^ in cytokinesis progression rather than in cytokinesis checkpoint.

Finally, we evaluated whether the Aurora-B-mediated phosphorylation of H2B-Ser32 is essential for H2B midbody recruitment. HeLa cells were transfected with a GFP-tagged wild-type H2B or with a non-phosphorylatable GFP-H2B-S32A mutant form. Similarly to wild-type H2B, the non-phosphorylatable H2B-S32A was present in telophase and midbody extracts (Fig. 4d). To exclude a possible contribution from contaminating nucleosomes, immunoblots were reacted with anti-p-Histone H2B-S14 Ab that, in the absence of apoptosis, specifically detects midbody-localized H2B [19] (Supplementary Figure S3d). Also in this condition, the H2B-S32A mutant was detected at midbody (Fig. 4e) indicating that S32 phosphorylation is dispensable for H2B recruitment.

Taken together, these data indicate that Aurora-B phosphorylates H2B at Ser32 and H2B-Ser32^P^ detection in mitosis follows Aurora-B and persists throughout cytokinesis. However, these data also indicate that additional Aurora-B-dependent event/s, besides phosphorylation on S32, are required for H2B recruitment.

### HIPK2 is recruited to midbody via Aurora-B-regulated central spindle components

**Fig. 5:**
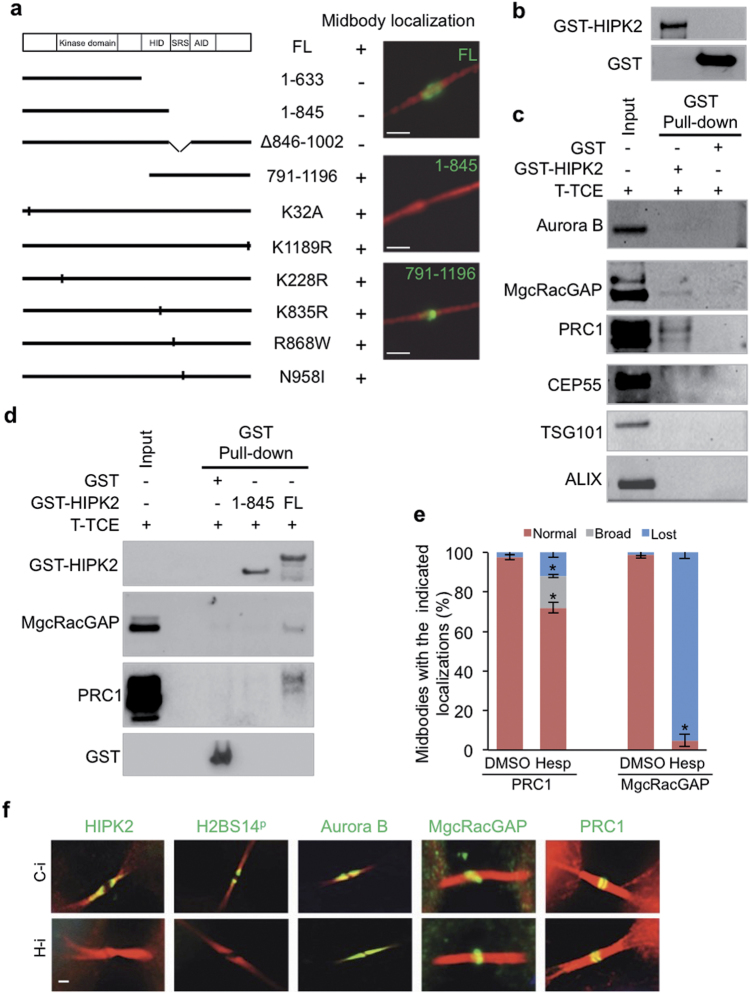
SRS region is essential for HIPK2 midbody localization and interaction with PRC1 and MgcRacGAP central spindle factors. **a** HeLa cells were transfected with vectors expressing tagged HIPK2-FL or relative derivative mutants. Numbers are referred to the current NCBI RefSeq of HIPK2 (NP_073577.3 and NP_034563.2). Cells were analyzed 24 h post-transfection by IF performed with anti-β-tubulin Ab to mark midbodies (red) in combination with opportune anti-tag Abs to visualize midbody localization of tagged HIPK2 forms. GFP-tagged proteins were visualized by autofluorescence. Midbodies of transfected cells were analyzed for the presence of tagged HIPK2 or relative mutants at the midbody in two independent experiments. Here reported is a schematic representation of the analyzed HIPK2 constructs with their localization at the midbody. Plus (+) indicates midbody localization in >90% of the cells; minus (−) indicates midbody localization in <5% of the cells. Left panel: schematic representation of HIPK2-FL with its domains (HID homeobox Interacting domain, SRS region containing speckle retention sequence, AID auto-inhibitory domain) and its deletion or point mutant forms. Right panels: representative images of midbodies with or without the indicated GFP-HIPK2 forms. Scale bar is 2 μm. **b**,** c** GST-HIPK2 and GST alone were produced in H1299 cells, purified by GST pull-down, analyzed by WB **b** and incubated with an equal amount of T-TCE (0.6 mg) obtained as in Fig. 4d. Proteins bound to GST-HIPK2 and GST were pulled-down and analyzed by WB with the indicated Abs. Representative WBs are shown **c**; input corresponds to 0.3% of T-TCE. **d** T-TCE from HeLa cells were obtained and incubated with the indicated GST-fusion proteins as in **b**,**c**. The presence of the indicated cytokinetic proteins was assessed by WB. Representative WBs are shown. Input corresponds to 0.3% of the T-TCE. **e** HeLa cells were treated with Hesperadin as in the Fig. 2. At least 100 midbodies for each condition have been scored and the percentages of midbodies with the indicated localization (i.e., normal, broad, and lost) are reported as mean ± SD of two independent experiments. *p < 0.05. **f** HeLa cells were transfected with HIPK2-specific stealth siRNAs (H-i) or negative control siRNA (C-i), fixed 4 days after transfection and analyzed by IF with anti-β-tubulin in combination with Abs against the indicated proteins. Scale bar is 1 μm

To get clues on the mechanism of Aurora-B-dependent recruitment of HIPK2 to midbody, we compared the subcellular distribution of HIPK2 full-length (FL) with a series of tag-HIPK2 mutants. Together with HIPK2 deletion forms, we tested a few point mutants including the tumor-associated N958I and R868W mutants, which have been shown to possess a reduced p53 activation capacity [35], the kinase-defective K228R [36, 37], the sumoylation-resistant K32A [38], the ubiquitylation-resistant K1189R [39], and the non-acetylatable K835R [40]. These experiments show that only the region encompassing amino acids 846–1002 of HIPK2, including the speckle retention signal (SRS), is necessary for midbody localization. In contrast, all the other mutants containing this region retain the capacity to localize at midbody (Fig. 5a), although its structure was slight altered (long, stretched bridges) by overexpression of each HIPK2-carrying vector, suggesting a mild toxic effect independent of the type of mutation.

The SRS region of HIPK2 is required for the physical interactions with many of its targets; [23] thus, we looked for cytokinesis factors that might interact with HIPK2. In order to enrich for cytokinesis factors, total cell extracts (TCEs) were obtained from telophase-synchronized HeLa cells and employed in GST-pull-down assay using purified GST-HIPK2(FL) (Fig. 5b). Such experiment confirmed that Aurora-B does not interact with HIPK2 (Fig. 5c, upper lane). In addition, we observed that abscission proteins (e.g., CEP55, TSG101, ALIX) that reach the midbody later in cytokinesis do not interact with HIPK2 (Fig. 5c, lower lanes). In contrast, MgcRacGAP and PRC1, two key factors in central spindle assembly [13, 14], are reproducibly present in the GST-HIPK2(FL) pull-downs (Fig. 5c, middle lanes) but not in the control GST.

Interestingly, Aurora-B indirectly regulates MgcRacGAP and PRC1 recruitment via their kinesin partners [14, 15]. However, depletion of MgcRacGAP or PRC1, or inhibition of their interaction, has been shown to deeply affect midbody formation [41, 42]. Therefore, to unveil a possible role of MgcRacGAP and/or PRC1 in the midbody recruitment of HIPK2, we followed indirect approaches.

First, we asked whether the HIPK2 region involved in midbody localization is also required for binding to central spindle proteins. As shown by GST-pull-down assays (Fig. 5d), the HIPK2 deletion mutant (1–845), lacking the C-terminal region necessary for midbody recruitment, is actually unable to bind MgcRacGAP and PRC1, suggesting a relationship between midbody localization and interaction with these central spindle factors.

Second, we assessed whether HIPK2 colocalizes with MgcRacGAP and PRC1 at midbody. Double IF analyses of early and late telophase showed that HIPK2 partially colocalize with the two central spindle factors (Supplementary Figure S4).

Third, we asked whether the Hesperadin-induced inhibition of HIPK2 midbody recruitment is also associated with inhibition of MgcRacGAP and PRC1 midbody localization. As shown in Fig. 5e, in the presence of Hesperadin the midbody localization of MgcRacGAP is strongly impaired and that of PRC1 is significantly affected, further supporting a correlation among these events. Thus, we evaluated whether the inhibition of HIPK2 midbody localization is consequent to or concomitant with that of MgcRacGAP and/or PRC1. To this aim, we assessed the midbody localization of the central spindle components upon HIPK2-specific RNA interference. As expected, HIPK2 depletion inhibits H2B-S14^P^ at midbody. However, we could not observe any alteration in the localization of MgcRacGAP, PRC1, and Aurora-B (Fig. 5f), indicating that HIPK2 recruitment temporally follows that of central spindle factors.

Taken together, these results suggest that Aurora-B indirectly recruits HIPK2 to the midbody through MgcRacGAP and PRC1.

### Impairment of Aurora-B-mediated activities on HIPK2 and H2B induces cytokinesis failure

We previously reported that HIPK2 depletion results in the loss of H2B-S14^P^ at midbody and in the accumulation of binucleated cells due to cytokinesis failure [19]. Analogous readouts are induced by Aurora-B inactivation [43]. Therefore, we asked whether the impairment of Aurora-B-mediated activities on HIPK2 and/or H2B might induce cytokinesis failure.

**Fig. 6:**
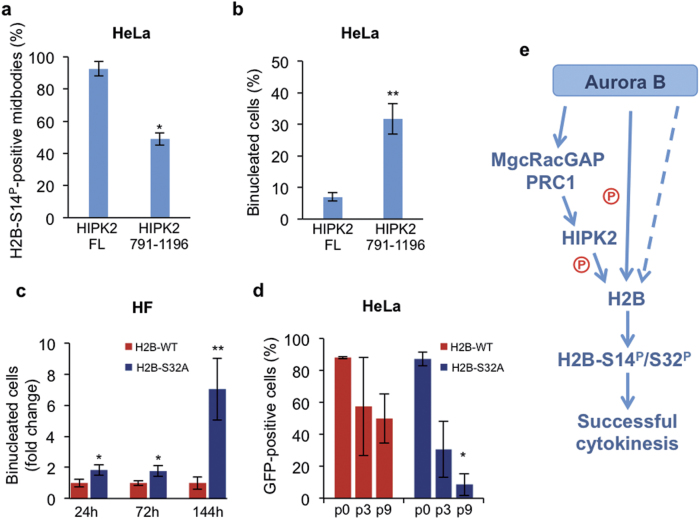
Impairment of Aurora-B-mediated activities on H2B and HIPK2 induces cytokinesis failure. **a**,** b** HeLa cells were transfected with vectors carrying the indicated GFP-HIPK2 forms and stained 24 h post-transfection with anti-p-Histone H2B-S14 and Hoechst. The percentages of midbodies positive for H2B-S14^P^ staining in the transfected populations **a** and the percentages of binucleated cells also in the transfected populations **b** are reported as mean ± SD of two different experiments. **c** HF were transfected with vectors carrying GFP-tagged H2B-WT or the non-phosphorylatable H2B-S32A derivative. Cells were stained with anti-β-tubulin Ab and Hoechst. The percentages of binucleated cells in the GFP-positive populations were evaluated at the indicated time-points post-transfection and reported as fold change relative to that of GFP-H2B-WT in two different experiments. *p < 0.05 and **p < 0.001 unpaired *t*-test. **d** HeLa cells were transfected with the same vectors as in **b** and treated with blastacidin 48 h after transfection to select stable transfected cells. The percentage of GFP-positive cells at 2 days (p0) after transfection, or 21 and 39 days after transfection (i.e., at the 3rd and 9th culture passage after blastacidin selection) is reported as mean ± SD of two different experiments. *p < 0.05 unpaired *t-*test, comparison between percentages of H2B-WT- and H2B-S32A-positive cells at each passage. **e** Schematic representation of Aurora-B activities on HIPK2 and H2B in cytokinesis. Aurora-B indirectly recruits HIPK2 via MgcRacGAP and PRC1 and directly phosphorylates H2B at Ser32. Additional Aurora-B-dependent event/s, besides phosphorylation on S32, are required for H2B recruitment. Dashed lines indicate still unknown mechanism/s. Phosphorylation of histone H2B at Ser32 by Aurora-B and Ser14 by HIPK2 contribute the successful cytokinesis

We inhibited midbody recruitment of the endogenous HIPK2 by sequestering its interactors, MgcRacGAP and PRC1. To this aim, HeLa cells were transfected with GFP-HIPK2(791–1196), the minimal C-terminal portion that still localizes at midbody (Fig. 5a), contains the region required to bind MgcRacGAP and PRC1, but lacks the kinase domain required to phosphorylate H2B at Ser14 and promote abscission [19]. We cannot directly demonstrate the absence of endogenous HIPK2 at midbody because anti-HIPK2 Abs recognize also the exogenous derivatives. However, we found that compared with HIPK2(FL), HIPK2(791–1196)-expressing cells have impaired ability to phosphorylate H2B-Ser14 (Fig. 6a) and perform successful cytokinesis, as shown by accumulation of binucleated cells (Fig. 6b).

Next, we assessed whether GFP-H2B-S32A, which cannot be phosphorylated by Aurora-B but can still localize at midbody, has functional consequences on cytokinesis. Compared with wild-type H2B, which does not induce significant effects and can be expressed for extended time, transfection of H2B-S32A resulted in accumulation of binucleated cells in human fibroblasts (HFs; Fig. 6c) and in the loss of H2B-S32A expression after a few passages in HeLa cells (Fig. 6d).

Overall, these results support a role for Aurora-B-mediated activities on HIPK2 and H2B midbody recruitment and in their contribution to faithful cytokinesis.

## Discussion

HIPK2 works as a haploinsufficient tumor suppressor and its inactivation increases tumorigenicity [44]. The tumor-suppressing activities of HIPK2 have been linked to cell protection against genome instability induced by different types of genotoxic stress, to inhibition of tumor invasion, multidrug resistance, and angiogenesis in hypoxia, and, more recently, to prevention of CIN as consequence of cytokinesis failure. While most of these activities depend on the nuclear functions of HIPK2 [44, 45], prevention of CIN relies, at least in part, on the midbody localization of HIPK2 and its target H2B, and their role in abscission for faithful cytokinesis [19, 20].

In this study, we investigated how HIPK2 and extrachromosomal histone H2B are recruited to the midbody. Stimulated by the recent observations that microRNAs can be involved in the formation of specific protein complexes [31], we asked whether RNA might be responsible for H2B or HIPK2 cytokinesis recruitment. We tested whether microRNA, single-strand RNA, double-strand RNA, or RNA/DNA hybrid might contribute to midbody recruitment of H2B or HIPK2. However, despite the good quality of our positive controls, we did not observe any defects in H2B or HIPK2 midbody localization, supporting the conclusion that nucleic acids are not involved in the cytokinetic distribution of the kinase and its histone target. Instead, we found that Aurora-B, one of the key mitotic kinases, is needed for the recruitment of both HIPK2 and H2B via two separate mechanisms (Fig. 6e).

Together with PLK1 and CDK1, Aurora-B is a key regulator of cell division. Aurora-B, as a component of the chromosomal passenger complex, localizes to the condensing chromosomes in prophase until the midbody in telophase, passing through the centromeres in metaphase and the central spindle in anaphase [11, 46]. Consistent with its spatio-temporal localization, Aurora-B regulates different mitotic and cytokinetic events promoting or delaying their progression, depending on specific events. Hence, Aurora-B kinase activity promotes chromosome condensation in mitosis and central spindle and midbody formation in cytokinesis. On the other side, Aurora-B inhibits mitosis progression to anaphase until all sister chromatid pairs are properly oriented (i.e., the spindle assembly checkpoint) and prevents abscission in cytokinesis when DNA remains at the cleavage site (i.e., the abscission checkpoint). Here, we found that Aurora-B directly binds and phosphorylates histone H2B at Ser32 in vitro. We observed that H2B-Ser32^P^ colocalizes with Aurora-B at the central spindle in anaphase A and persist throughout the subsequent steps, until abscission. Of relevance, this behavior was detectable at each cytokinesis and was independent from the presence of aberrant DNA at the cleavage plane, thus indicating that H2B-Ser32 phosphorylation by Aurora-B promotes, rather than delays, cell division. In addition, our results indicate that histone H2B is temporally recruited for cytokinesis before HIPK2 and this is consistent with the observation that H2B is present at the midbody also in Hipk2-/- MEFs [19].

As assessed by IF with anti-histone H2B Ab that recognizes the histone independently of its posttranslational modifications, inhibition of Aurora-B kinase activity by Hesperadin or ZM-447439 blocks H2B recruitment. However, the expression of a non-phosphorylatable H2B-S32A mutant showed that Ser32 phosphorylation is dispensable for H2B recruitment because the mutant can still localize at midbody and be phosphorylated at Ser14 by HIPK2. Taken together, these results suggest that other Aurora-B-dependent activities are necessary for the recruitment of H2B. Indeed, based on the complex network of interactions among the microtubule modulators and their regulators during the assembly of the central spindle and midbody [13], this result is not particularly surprising. However, as discussed below, H2B-Ser32^P^ is functionally required for faithful cytokinesis.

We obtained direct and indirect evidence suggesting that HIPK2 is recruited to the midbody through the Aurora-B-regulated central spindle factors MgcRacGAP and PRC1. Of relevance, during cytokinesis, HIPK2/PRC1 colocalization was previously reported by Hofmann et al. [45]. An important test for the functional significance of these interactions would have been the evaluation of localization epistasis. However, the key role of PRC1 and centralspindlin as hub factors limited our analyses to the evaluation of the proper localization of central spindle factors upon depletion of HIPK2, while the opposite (PRC1 or MgcRacGAP depletion) could not be informative for HIPK2 midbody localization [41, 42]. At this point, we do not know whether there is a direct binding between HIPK2 and central spindle factors. However, we showed that the HIPK2 region required for interaction with MgcRacGAP and PRC1 (the SRS containing region) is also required for midbody localization. Interestingly, the SRS region, besides mediating the interactions with most of the non-homeotic factors, is also involved in the subnuclear localization of HIPK2 into speckled structures [23, 47].

HIPK2 activity and stability are controlled by different posttranslational modifications [48]. We previously showed that the kinase-defective HIPK2-K228R mutant, which is largely hypophosphorylated [21, 22], is still able to localize at the midbody but it is not able to phosphorylate H2B and does not rescue the abscission failure in Hipk2-/- MEFs [19]. Here, we confirmed this observation and extended the analysis to other posttranslational modification-defective mutants, including sumoylation-defective, acetylation-defective, ubiquitylation-resistant, and tumor-associated point mutants. All of these mutants retain the ability to localize at midbody indicating that the posttranslational modifications relevant for HIPK2 activity in stressing conditions are dispensable for the localization at midbody during cytokinesis. Altogether, these results support the conclusion that, in contrast to stressing conditions, the recruitment at midbody of HIPK2 depends on protein/protein interactions rather than posttranslational modifications.

Aurora-B is frequently overexpressed in human cancers and several small molecule inhibitors have been developed and tested in preclinical studies and clinical trials [49, 50]. Consistent with the spatio-temporal localization of Aurora-B, these inhibitors mainly act in mitosis by silencing the spindle assembly checkpoint and leading to chromosome missegregation, cytokinesis failure, and polyploidization [51, 52]. Aurora-B overexpressing tumor cells are particularly sensitive to these effects and eventually undergo cell death. However, proliferating normal cells are also significantly affected resulting in meaningful clinical toxicity [53]. It has been shown that targeting mitotic exit without affecting the spindle assembly checkpoint might have a larger therapeutic window [54]. Yet, such effect cannot be obtained by directly inhibiting the kinase activity of Aurora-B. Thus, a block of the interactions with its cytokinesis partners has been proposed [55]. Here, we show that among different cytokinesis functions, Aurora-B separately recruits HIPK2 and H2B to the midbody and doing so contributes to faithful cytokinesis. Indeed, inhibition of these Aurora-B recruitment activities, by expressing the non-phosphorylatable H2B-S32A mutant or the minimal C-terminal portion of HIPK2 that binds and sequesters MgcRacGAP and PRC1, results in accumulation of binucleated cells. These data confirm a key role for HIPK2 and H2B in cytokinesis and offer the possibility of testing HIPK2 and extrachromosomal histone H2B as therapeutic targets for inhibition of cell division.

## Materials and methods

### Cell culture and reagents

HeLa (kind gift of C. Passananti), H1299 (kind gift of G. Blandino), U2OS (kind gift of F. Moretti), hTERT-immortalized HF [56], HCT116 parental or *DICER*-defective (kind gift of B. Vogelstein) were cultured at 37 °C and 5% CO_2_ in Dulbecco’s modified Eagle’s medium GlutaMAX supplemented with 10% fetal bovine serum, penicillin/streptomycin (Life Technologies) and routinely tested for mycoplasma contamination. Cells were transfected using Lipofectamine LTX and PLUS reagent for plasmid DNA and RNAiMAX for small interfering RNAs (siRNAs; Life Technologies). HIPK2-specific RNA interference was performed as described [19]. Kinase inhibitor treatments were performed on unsynchronized, proliferating cells. Hesperadin (Selleckchem) and ZM-447439 (Signalchem) were used to inhibit Aurora-B and Bi 2536 (AbMole) to inhibit PLK1. Solvent dimethylsulfoxide (DMSO; Sigma-Aldrich) was used as control.

### Enrichment in telophase, midbody isolation, and RNase treatments

HeLa cells were enriched in telophase by treatment with nocodazole (Sigma-Aldrich; 100 ng/ml for 4 h) followed by mitotic shake-off and nocodazole wash out. Collected cells were plated on poly-l-lysine-coated coverslips and incubated to reach different cytokinesis stages. Midbodies were isolated as described [19, 57] and extracted in buffer (50 mM Tris-HCl pH 7.4, 600 mM NaCl, 0.1% sodium dodecyl sulfate (SDS), 0.5% NP40, 1 mM DTT, 5 mM EDTA) supplemented with protease- and phosphatase-inhibitor mix (Roche).

In vivo RNase A treatment was adapted from Francia et al. [31]. HeLa cells were plated onto poly-l-lysine-coated coverslips and UVB irradiated. One hour later, cells were permeabilized with increasing percentage of Tween20 (0.5, 0.75, 1, 1.5 or 2%) in phosphate-buffered saline (PBS) for 10 min at room temperature (RT). RNase A treatment was carried with 1 mg/ml ribonuclease A from bovine pancreas (Roche) in PBS for 25 min at RT; PBS alone or was used as control. Next, cells were fixed in 2% formaldehyde and immunoreacted with anti-53BP1 Ab (Abcam). Mitotic enriched HeLa cells (45 min post-nocodazole wash out) were permeabilized with 0.75% Tween20 and treated with PBS, RNase A (Roche), RNase III (25U; Ambion), RNase H (25U, Ambion), or DNase I (25U; Ambion), fixed with 2% formaldehyde for 10 min or cold methanol 100% for 5 min at −20 °C.

### Western blotting

TCEs were prepared in lysis buffer (50 mM Tris-HCl pH 8.0, 150 mM NaCl, 0.5% sodium-deoxycholate, 0.1% SDS, 1% NP40 and 1 mM EDTA) supplemented with protease inhibitor mix (Roche) and Halt Phosphatase Inhibitor Cocktail (Life Technologies). Proteins were resolved by SDS-polyacrylamide gel electrophoresis (PAGE) using NuPAGE® Novex Bis-Tris Gels (Life Technologies), transferred onto nitrocellulose membranes (Bio-Rad), and analyzed with the required Abs. Horseradish peroxidase (HRP)-conjugated goat anti-mouse and anti-rabbit secondary Abs (Bio-Rad) were used. Immunoreactivity was determined by ECL-chemioluminescence reaction (Amersham). The following Abs were employed: anti-TSG101, anti-cep55, anti-MgcRacGAP1, anti-p-histone-H2B-S32, and anti-Aurora-B (Abcam); anti-PRC1, anti-Alix, anti-PCNA, and anti-GST (Santa Cruz); anti-GFP (Roche); anti-p-histone-H2B-S14 (Cell Signaling).

### IF microscopy

Cells were seeded onto poly-l-lysine-coated coverslips, fixed in ice-cold methanol or 2% formaldehyde, washed three times in PBS, permeabilized in 0.25% Triton X-100 in PBS for 10 min, blocked in 0.2% Triton X-100, 5% bovine serum albumin in PBS for 60 min before the required primary Abs were applied. The following Abs were employed: anti-Aurora-B (BD-Bioscience); anti-ECT2, anti-PRC1, anti-MKLP1, and anti-PLK1 (Santa Cruz); anti-HIPK2 (rabbit polyclonal Ab [19]); anti-p-histone-H2B-S14 (Cell Signaling); anti-p-histone-H2B-S32, anti-H2B, anti-53BP1, and anti-MgcRacGAP1 (Abcam); anti-β-tubulin-Cy3 and anti-α-tubulin-FITC (Sigma-Aldrich); secondary 488- or 594-conjugated Abs (Life Technologies). DNA was marked with Hoechst 33342 (Sigma-Aldrich) or Red-Dot2 far-red (Biotium). Cells were examined under an Olympus BX53 microscope using a cooled camera device (ProgRes MF) and with confocal microscope Zeiss LSM510-Meta.

### Expression vectors and recombinant proteins

The following expression vectors were employed: pEGFP-c2, pEGFP-HIPK2-FL and its derivative deletion and point mutants pEGFP-HIPK2-1-633; pEGFPHIPK2-1-845; pEGFP-HIPK2-delta846-1002; pEGFP-HIPK2-791-1196; pEGFP-HIPK2-K228R, pEGFP-HIPK2-K1189R [39]; pLUCX-HA-HIPK2-R868W and pLUCX-HA-HIPK2-N958I [35] kind gift of I. Kitabaiashi; pcDNA3-Flag-HIPK2-K32 and pcDNA3-Flag-HIPK2-K835R [40] kind gift of L.M. Schmidtz, pBOS-GFP-H2B (BD PharMingen). pBOS-GFP-H2B-S32A mutant was obtained by site-directed mutagenesis using QuickChange kit (Stratagene) and analyzed by sequencing. To gain high levels of GST, GST-HIPK2-FL or its derivatives, H1299 cells were infected with vTF7-3 followed by transfection with pcDNA3-eGST, pcDNA3eGST-HIPK2 or its derivatives, as described [22]. Recombinant GFP and GFP-HIPK2 were produced in U2OS cells by transfection of pEGFP-c2 or pEGFP-HIPK2, as described [58]. GST-Aurora-B (A2108) and MBP (M1891) are from Sigma-Aldrich, His-H2B (Ag7811) from Proteintech.

### GST pull-down and binding assay

GST-fusion proteins were purified from TCEs using Glutathione-Sepharose 4 Fast Flow (GE Healthcare), incubated for 2 h at 4 °C with glutathione sepharose beads, pulled-down, washed three times with nondenaturing lysis buffer, and used as bait in binding assays. For H2B and Aurora-B binding assays, GST-fusion proteins were incubated with 500 ng of recombinant His-H2B (Proteintech) in 50 mM Tris-HCl pH 7.5 at two salt concentrations, 150 and 250 mM NaCl. For T-TCE binding assays, GST-fusion proteins were incubated with 0.6 mg of T-TCE in binding buffer (50 mM Tris-HCl pH 7.5, 200 mM NaCl, 0.5% NP40). For HIPK2 and Aurora-B binding assays, GFP or GFP-HIPK2 were purified as described [58]. TCEs were prepared 24 h post-transfection of U2OS cells. Supernatants obtained after TCE centrifugations were incubated overnight with anti-GFP Ab-sepharose beads (Abcam) at 4 °C. GFP-proteins were incubated with GST-Aurora-B in 50 mM Tris-HCl pH 7.5 at two salt concentrations (150 and 250 mM NaCl). Bound proteins were analyzed by WB.

### Kinase assay and mass spectrometry

Kinase assays were performed incubating 150 ng of GST-Aurora-B in kinase buffer (Hepes 20 mM pH 7.5, 1 mM DTT, 10 mM MgCl_2_ and 1 mM EGTA) in the presence of γ-^32^P-ATP (Perlkin-Elmer, BLU502Z250UC) at 30 °C for 30 min. MBP, His-H2B, or GST-HIPK2-K228R were used as substrates. GST-HIPK2-K228R alone was used to evaluate the autophosphorylation level of the defective mutant. Proteins were resolved by SDS-PAGE and phosphorylation analyzed by autoradiography.

Cold kinase assays were performed with 2 μM ATP (Roche) for 1 h and phosphorylated H2B analyzed by WB or MS. For MS, H2B was in-gel digested with trypsin, chymotrypsin, AspN or GluC and peptides analyzed by liquid chromatography MS using an Ultimate 3000 HPLC (DIONEX) connected on line with a linear Ion Trap (LTQ, Thermo). MS/MS and MS/MS/MS were acquired in order to search for phosphopeptides carrying mass increase and neutral losses, respectively. Tandem mass spectra were analyzed by Proteome Discoverer software (version 1.4, Thermo Electron) searching.

### Statistics

All data are presented as mean ±S.D. (standard deviation). The* p*-values were derived from unpaired two-tailed *t*-tests using GraphPad Prism software. The *p*-values < 0.05 were considered significant. In all, 80 midbodies and 1000 cells for each condition were scored, unless otherwise stated.

## Electronic supplementary material

Supplementary Figures and Legends(PDF 4449 kb)
