# Supplementary material for: HIPK2 and extrachromosomal histone H2B are separately recruited by Aurora-B for cytokinesis
Source: Oncogene. 2018 Mar 22;37(26):3562–74. doi: 10.1038/s41388-018-0191-6 (PMC6021368; doi:10.1038/s41388-018-0191-6)
Supplement: Supplementary file 1 — Supplementary Figures and Legends(PDF 4449 kb) [file 41388_2018_191_MOESM1_ESM.pdf]

SUPPLEMENTARY FIGURES and LEGENDS

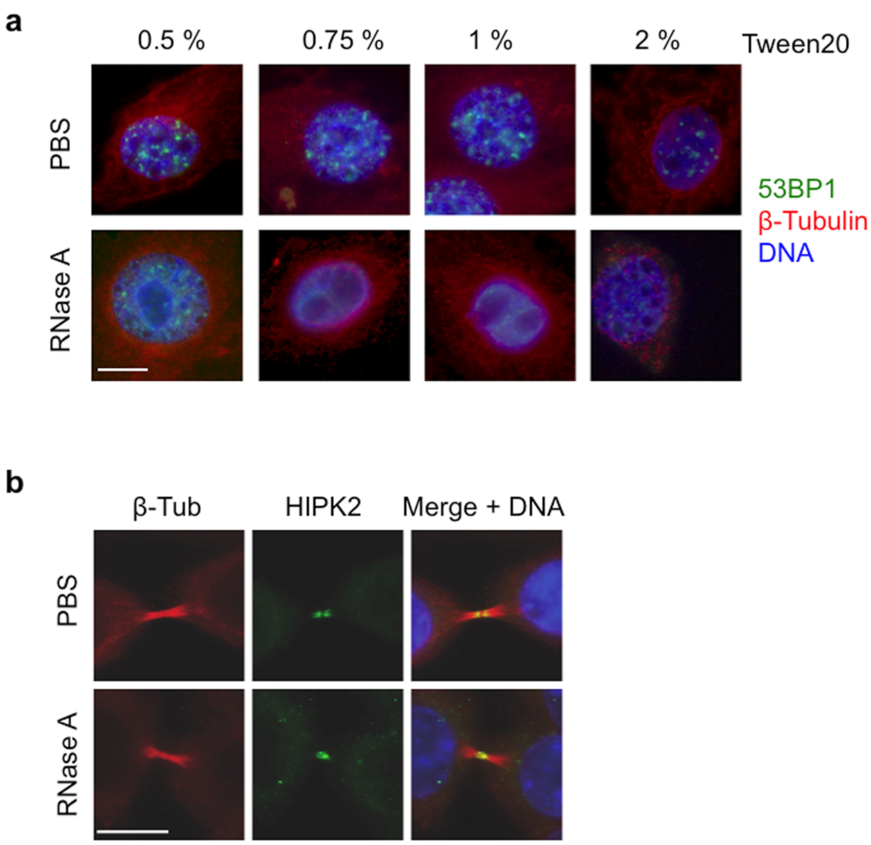

Supplementary Figure S1. RNase A treatment validation and HIPK2 localization analyses. (**a**) In order to reproduce the RNase A-treatment performed by Francia et al.,[31] HeLa cells were irradiated with UVB 0.075 J/cm^2. After one hr, cells were permeabilized with 0.5, 0.75, 1, or 2% Tween20 for 10 min and treated with RNase A (1 ml of 1 mg ml^{-1}) or PBS for 25 min, fixed with 2% formaldehyde and analyzed for the disappearance of 53BP1 foci (Green). Tubulin was marked with anti-β-tubulin Ab (red) and DNA was counterstained with Hoechst (blue). In HeLa cells, permeabilization with 0.75% Tween20 is sufficient to assure loss of 53BP1-positive foci. (**b**) HIPK2 localizes independently from RNase A treatment. Mitotic enriched HeLa cells (45 min post-mitotic shake-off and nocodazole wash out) were permeabilized with 0.75% Tween20 and treated with PBS or RNase A, and assessed by IF for the presence of HIPK2 (green), tubulin (red), and DNA (blue). Each midbody visualized (n>50 in two independent experiments) was positive for HIPK2 staining. Scale bar is 10μm.

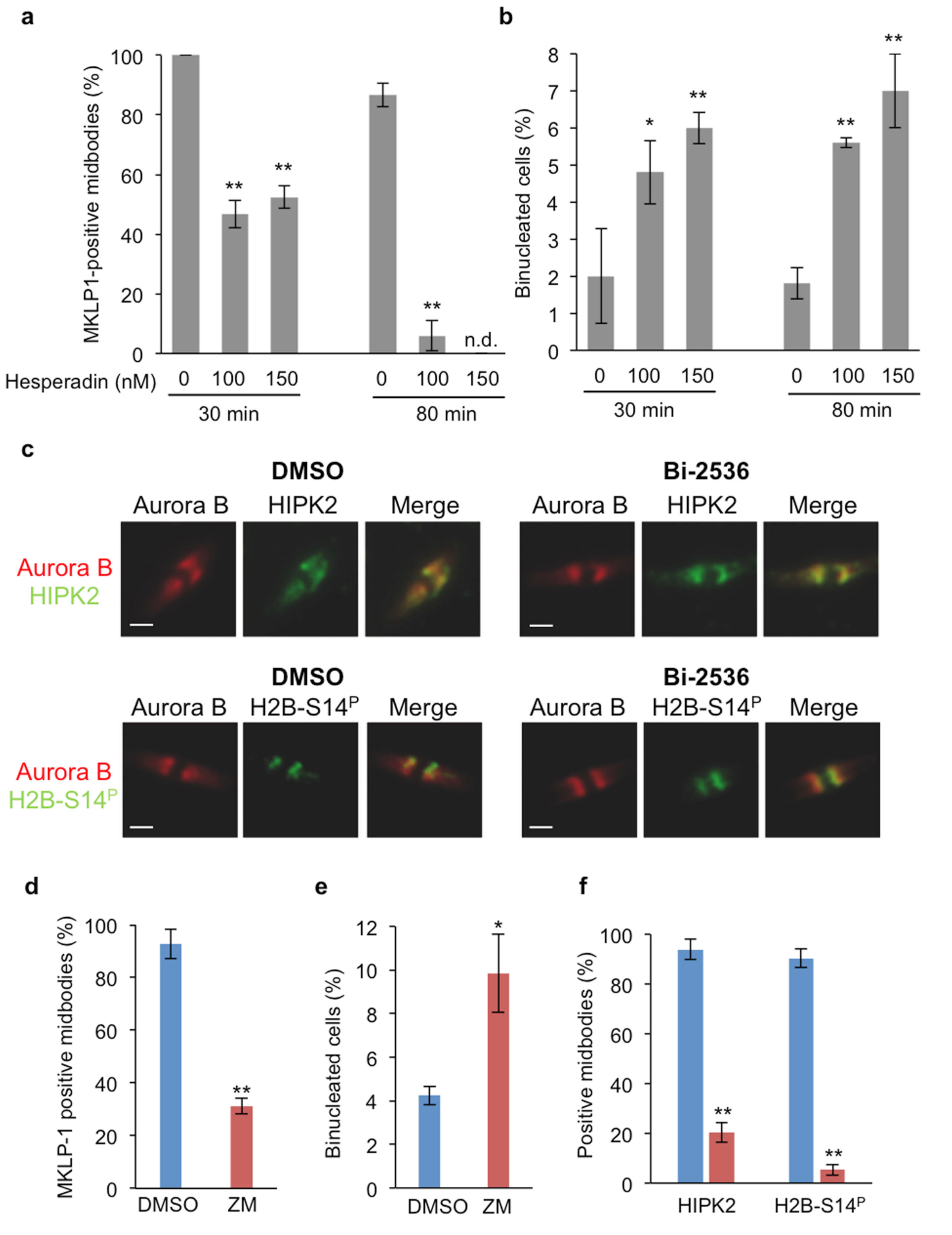

Supplementary Figure S2. Evaluation of midbody-specific effects of Aurora-B inhibitors. (**a-b**) The proper concentration and incubation period of Hesperadin on proliferating HeLa cells were empirically determined by assessing the reduction of MKLP1 midbody localization (*i.e.*, an Aurora-B-recruited kinesin) and the accumulation of binucleated cells (*i.e.*, a functional sign of abscission failure). To this

aim, unsynchronized HeLa cells were treated with increasing concentration of Hesperadin and analyzed at the indicated time-points post-treatment; solvent DMSO was used as control. After treatment, cells were fixed and stained with anti-MKLP1 and anti-β-tubulin Abs. (**a**) 80 midbodies for each condition have been scored in two independent experiments; the percentages of midbodies positive for MKLP1 staining are reported as mean ± SD. (**b**) 1,000 cells for each condition have been scored and the percentages of binucleated cells are reported as mean ± SD of two independent experiments. (**c**) HeLa cells were treated with Bi-2536 as in Figure 2a. After treatment, cells were fixed and stained with the indicated Abs. Representative images are reported. Scale bar is 1μm. (**d-f**) Unsynchronized HeLa cells were treated with 2μM ZM-447439 (ZM) and analyzed as in **a** and **b**, 80 min post-treatment. The percentages of MKLP1-positive midbodies (**c**) and of binucleated cells (**d**) are reported as mean ± SD of three independent experiments. (**e**) Unsynchronized HeLa cells were treated as in **c** and analyzed for the localization of HIPK2 and H2B-S14^{P} at midbody. IF analyses were performed with rabbit anti-HIPK2 and anti-p-Histone H2B-S14 Abs in combination with anti-β-tubulin Ab. DNA was visualized with Hoechst. 80 midbodies for each condition have been scored and the percentage of midbodies positive for the indicated proteins is reported as mean ± SD of three independent experiments. * $p < 0.05$ and ** $p<0.001$ unpaired t-test. n.d.=not determinable.

a

10 20 30 40 50

MPEPAK**S**APA PKKG**S**KKAV**T** KAQKKDGKKR KR**S**RKE**S**Y**S**I YVYKVLKQVH

60 70 80 90 100

PD**T**GI**SS**KAM GIMN**S**FVNDI FERIAGEA**S**R LAHYNKR**ST**I **TS**REIQ**T**AVR

110 120

LLLPGELAKH AV**S**EG**T**KAV**T** KY**TSS**K

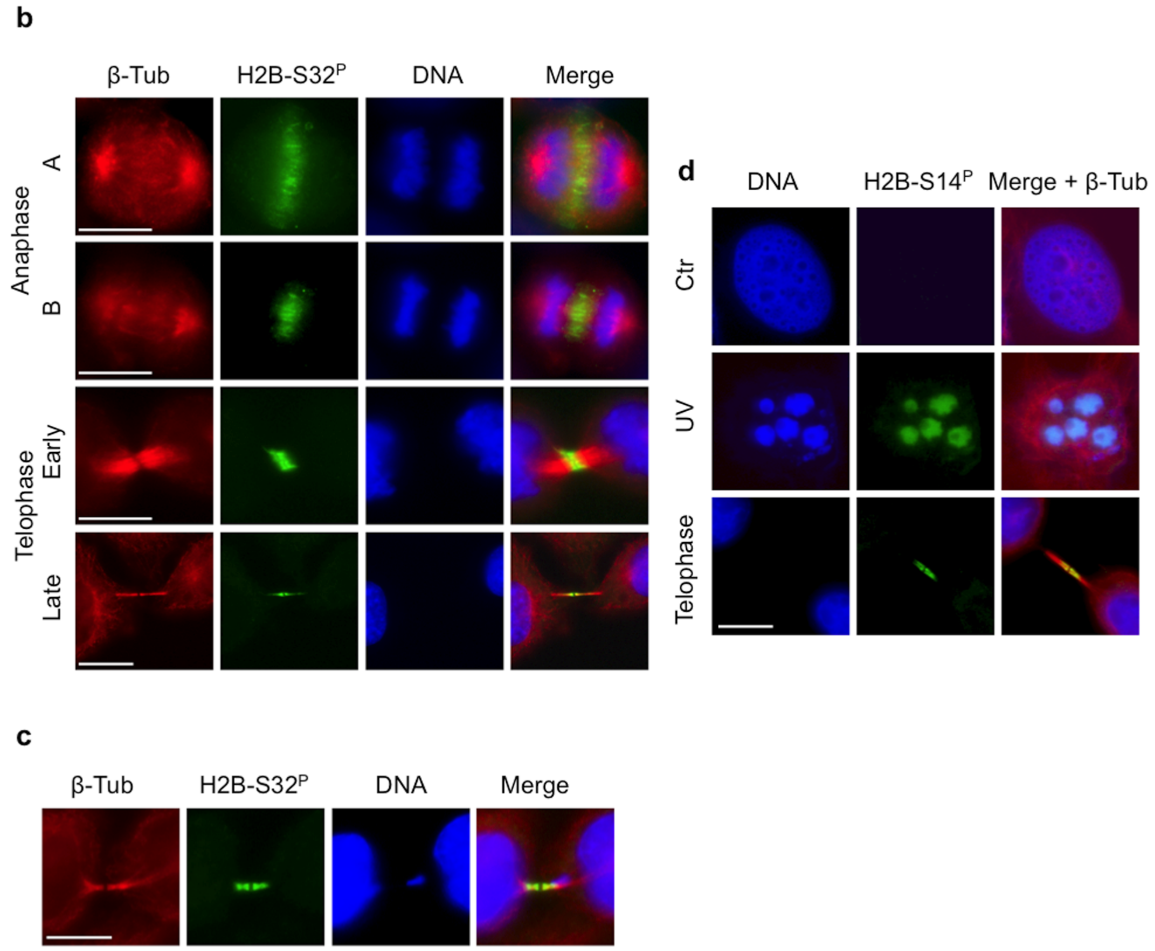

Supplementary Figure S3. Analyses of Histone H2B phosphorylation. (**a**) Histone H2B aminoacid sequence; all serine (S) and threonine (T) are indicated in bold. The putative S and T residues belonging to the Aurora-B consensus sequence, analyzed by GPS 3.0 and KinasePhos 2.0, are underlined in green. The Ser32 phosphorylated by Aurora-B kinase, as described in Figure 4a, is indicated in red. The sequence analyzed by MS is highlighted in gray. All the putative residues detected in this region by MS were found only in the non-phosphorylated form. (**b**) H2B-Ser32^{P} localizes in different stage of cell division, from anaphase A to late telophase. Representative images of proliferating HeLa cells are reported and stained with anti-p-Histone H2B-S32 Ab (green), anti β-tubulin (red) and Hoechst

(blue) to mark DNA. (**c**) H2B-Ser32^{P} localizes at the midbody independently of the presence of chromosomal bridges. Representative images of HeLa cells stained as in **b** are reported. (**d**) Representative images of histone H2B-Ser14^{P} in HeLa cells control (Ctr) or UVB-irradiated to induce apoptosis. As expected, the DNA is negative for the H2BSer14^{P} staining in the nucleus of control cells (upper panels) and on the contrary, apoptotic DNA is positive (middle panels). In telophase the anti-p-histone H2B-S14 Ab specifically stains the extrachromosomal histone H2B that localizes at the midbody (lower panels). Scale bars are 10μm.

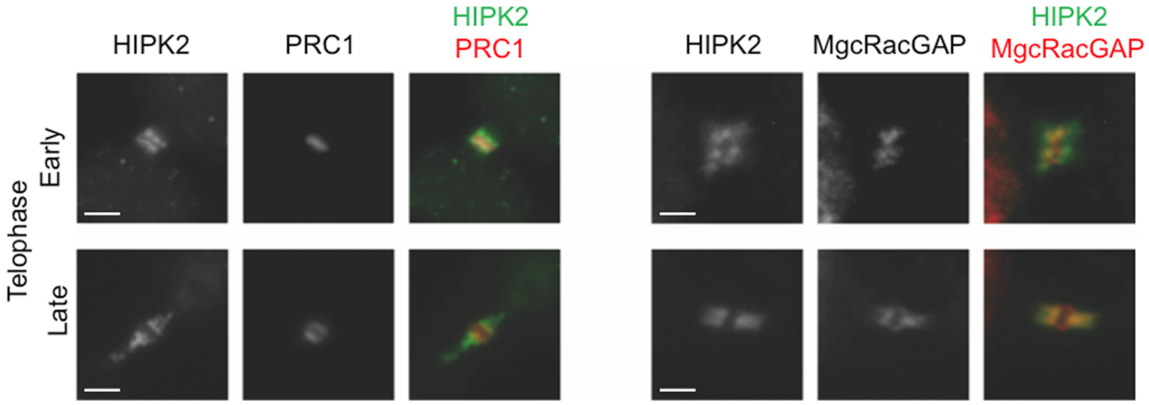

Supplementary Figure S4. Analyses of HIPK2 and central spindle factors localization. HeLa cells were fixed and stained with anti-HIPK2 (green) in combination with anti-PRC1 (red) or anti-MgcRacGAP (red). Hoechst was used to stain DNA. Representative images of midbodies are reported showing that HIPK2 partially overlaps with the two central spindle factors during early telophase, while, after midbody maturation the three proteins mainly localize in different zones (*i.e.* HIPK2 at the flanking zone, PRC1 at the dark zone and MgcRacGAP at the bulge zone). Scale bar is 2μm.
